# Nanotransition Materials (NTMs): Photocatalysis, Validated High Effective Sorbent Models Study for Organic Dye Degradation and Precise Mathematical Data’s at Standardized Level

**DOI:** 10.3390/nano8030134

**Published:** 2018-02-27

**Authors:** Farheen Khan, Rizwan Wahab, Mohamed Hagar, Rua Alnoman, Lutfullah, Mohd Rashid

**Affiliations:** 1 Department of Chemistry, Faculty of Science, Taibah University, Yanbu 42353, Saudi Arabia; Mohamedhaggar@gmail.com (M.E.H.); Rua-b-n@live.co.uk (R.A.); 2 Department of Zoology, College of Science King Saud University, Riyadh 11451, Saudi Arabia; 3 Al-Jeraisy, Chair for DNA Research, Department of Zoology, College of Science, King Saud University, Riyadh 11451, Saudi Arabia; 4 Department of Chemistry, Faculty of Science, Alexandria University, Alexandria 21321, Egypt; 5 Department of Chemistry, Aligarh Muslim University, Aligarh UP 202002, India; lutfullah786@gmail.com; 6 Saraswati Devi Post Graduate College, Khadda Bazar, Kushinagar UP 274802, India; mohammadrashid87@gmail.com

**Keywords:** CuONP synthesis, methylene blue (MB) dye, photocatalytic degradation, kinetic and equilibrium isotherm, thermodynamics, error analysis

## Abstract

The present work describes the synthesis of copper oxide nanoparticles (CuONPs) via a solution process with the aim of applying the nano-adsorbent for the reduction of methylene blue (MB) dye in alkaline media. These NPs were characterized via Field emission scanning electron microscopy (FE-SEM), X-ray diffraction, high-resolution Transmission electron microscopy (TEM), and ultra violet UV-visible spectroscopy to confirm their morphology and crystalline and optical properties in order to design an adsorption-degradation process. The photocatalytic CuONPs exhibited dynamic properties, great adsorption affinity during the chemisorption process, and operated at various modes with a strong interaction between the adsorbent and the adsorptive species, and equilibrium isotherm, kinetic isotherm, and thermodynamic activities in the presence of UV light. All basic quantities, such as concentration, pH, adsorbent dose, time, and temperature, were determined by an optimization process. The best-fitted adsorption Langmuir model (*R*^2^ = 0.9988) and performance, including adsorption capacity (350.87 mg/g), photocatalytic efficiency (90.74%), and degradation rate constant (*K*s = 2.23 ×10^−2^ min^−1^), illustrate good feasibility with respect to sorption-reduction reactions but followed a pseudo-second-order kinetic on the adsorbent surface, reaching an equilibrium point in 80 min. The thermodynamic analysis suggests that the adsorption reaction is spontaneous and endothermic in nature. The thermodynamic parameters such as enthalpy (∆H°), entropy (∆S°), and Gibbs free energy (∆G°) give effective results to support a chemical reduction reaction at 303 K temperature. The equilibrium isotherm and kinetic and thermodynamic models with error function analysis explore the potential, acceptability, accuracy, access to adsorbents, and novelty of an unrivaled-sorption system.

## 1. Introduction

The metal oxide nanomaterials have multidisciplinary applications in basic and applied sciences due to their large surface area, small size, higher surface lattice energy, high thermal reactivity, etc. It illustrates their beneficial purposes in processes such as adsorption, catalysis, energy conversion storage, optoelectronics, and drug delivery systems [1,2,3,4,5,6]. The nanomaterial powders are employed in industry in various ways, connecting the physicochemical, biological, engineering, and health sectors [7]. Many kinds of materials are used for the removal of dyes, via adsorption processes, from agricultural waste material, metals, organic compounds, textile wastes, etc. [8,9,10]. To remove organic dyes from water, various procedures have been applied but are costly and do not give effective results; the metal oxide (MO) nanomaterials (NMs) are the best alternative and the most effective material, having participated in the removal of hazardous/toxic dyes in water. The MONMs have properties that overcome the complications of removing hazardous/toxic dyes from water and the environment through reduction or degradation processes [11,12]. A series of methods have been designed for the removal of dyes, such as adsorption, ion exchange, filtration/coagulation, ozonization, Fenton reagent, photocatalytic reactions, aerobic degradation, anaerobic degradation, and biosorption. All these methods belong to three categories: physical, chemical, and biological methods. In comparison to all these methods, the adsorption method is the most popular, most effective, simplest, cheapest, and is a routinely used technique on larger application scales. Generally, adsorption techniques provide selective and easy-to-use sorbents and cleanup medium, are simple, and have easy sample preparations for the isolation/removal of toxic substances. These reasons and its small risk of errors are the main advantages of adsorption techniques, which are traditionally used in laboratories. As per scientific depiction, adsorption processes follow equilibrium and kinetics processes; both processes provide simultaneous advantage: first, the kinetic rate of the reaction of the overall adsorption mechanism and second, adsorption-desorption equilibrium analysis performance at equivalent, stable and constant time in the presence of all optimized parameters. In the adsorption system, the fitness of the four kinetic models (Lagergren pseudo-first-order, Ho and McKay pseudo-second-order, Elovich model, and intraparticle diffusion) and the four equilibrium isotherm models (Freundlich, Langmuir, Dubinin–Radushkevich, and Temkin) are based on linear regression methods that correspond to the feasibility of a nanomaterial system combined with methylene blue (MB) ions on the adsorbent’s surface [13,14]. Various types of engineered nanoparticles, such as zinc, magnesium, iron, aluminum oxide, cerium oxide, and zirconium oxide, are being extensively utilized for the removal of organic wastes [15]. Other versatile nanostructures of MOs, the CuO nanostructures, which arise in the d-block transition metal series, have unique properties (such as catalytic, antimicrobial, and antifungal) and various applications (such as in industry, electronics, optoelectronics, sensors, and biomedical applications). The requirement to clean, freshen, and save the environment from organic carcinogenic compounds, dyes and more hazardous environmental contaminants [16,17] can be aided via photocatalytic CuONMs [18]. CuO has various physical properties, such as p-type semiconductor, an energy band gap of 1.21–1.5 eV, and good quantum efficiency. It has been tested as a photocatalyst for the deactivation of organic molecules/dyes, which it decomposes or deactivates mainly via a photocatalytic process that is more impulsive under UV-visible light [19]. Numerous physicochemical methods can be applied to synthesize the nanostructures of copper, such as plasma-enhanced chemical vapor deposition (PECVD), surface mechanical attrition treatment (SMAT), chemical vapor deposition (CVD), metal-organic chemical vapor deposition (MO-CVD), etc. The chemical methods such as solution, sol-gel, hydrothermal, combustion, and spray pyrolysis [20,21,22,23,24,25] can provide large quantities of nanostructures. Organic dyes are highly hazardous to the human body and cause a series of diseases, such as hypertension, precordial pain, mental confusion, nausea, profuse sweating, methemoglobinemia, skin staining, dizziness, headache, Alzheimer, anemia, and eye-burn problems [26,27,28,29,30,31]. To solve this problem and for a healthy environment, it is very important to understand and study the toxic substances (dyes) and their relations with biological species [32,33,34,35]. Toward this goal, several parameters have been applied (such as the concentration of dye, duration of toxic effects, control dyes, and applied technique) to regulate their toxic effects; therefore, from the fitness consideration of these points, the analytical technique is fully able to detect, identify, quantify toxic dyes and organic substances [33,34]. Due to its fast, simple, and cheap methodology, it provides satisfactory results for colored and colorless routine laboratory samples analyzed via UV-visible spectrophotometer [36]. A few amount of nanomaterials has the capability to degrade organic dyes into small fragments and can remove the pollution [32,33,36]. Different types of copper precursors (such as copper nitrate hexahydrate, copper chloride, and copper sulfate) have been utilized to degrade methylene blue (MB) and rhodamine B (RhB) dyes in an aqueous solution [37]. In another report, including the nanoparticles, the polymer nanocomposites (chitosan/polyvinyl alcohol/copper oxide nanorods, CS/PVA/CuO) were utilized as an adsorbent for the removal of Pb(II) ions from an aqueous environment [38]. In this continuation, the inorganic nanocomposite of (CuO/MCM-41 nano composite) CuO, which exhibits a high potential in the treatment of wastewater [14], was used as an adsorbent for the removal of cationic dyes. Towards this direction, researchers have done and explained the adsorption capacity of different types of nanomaterials (NMs) for the removal of heavy metals and dyes [39,40,41,42] and have employed and discussed the adsorption capacity, efficiency, isotherms, and kinetic parameters.

The thermodynamic parameters (ΔH°, ΔG°, ΔS°, and ΔE°) and kinetic models give more detail about the consequences of NMs. The thermodynamic and kinetic studies describe better the relationship between NMs and other chemical species with good adherence or adsorption properties. An adsorption kinetic study performed in an aqueous solution with constant temperature provides valuable information related to the fixed number of sites on the adsorbent and the adsorbate material. To evaluate the consequences of dyes in wastewater, various technologies have been applied: biological, chemical, and physical. These methods are very useful for environmental purification. As per the detailed literature survey, various adsorption studies have been performed for organic, inorganic, hybrid, and composite materials, which have included isotherm, kinetics, thermodynamic studies for assessing the adsorption and desorption. Basically, a heterogeneous system favors adsorption techniques, which require more than one ingredient, provide suitability and efficiency and easily adopt the study of equilibrium and kinetics [39,40,41,42].

The present work describes first the synthesis of copper oxide nanoparticles, referred to as CuONPs, using a solution process, and then the characterization of CuONPs using XRD (X-ray diffraction pattern), FE-SEM (Field emission scanning electron microscopy), and TEM (Transmission electron microscopy). Besides this, the main objective of our work is to investigate the parameters that influence the initial solution pH, adsorbent dose, initial MB dye concentration, and temperature. The study provides information related to collective results such as kinetic sorption study analyses via pseudo-first- and second-order, Elovich model and intraparticle diffusion mechanism (IPDM). The equilibrium study was better fitted to the Langmuir and Freundlich models than the Hasley, H–J, Temkin, and D–R models. The thermodynamic study of the heat of adsorption at a constant temperature gives the values of the thermodynamic parameters (ΔH°, ΔG°, ΔS°, ΔE°). Also evaluated and included here are the different error analyses: the residual root mean square error (RMSE), the Chi-square test, sum of the squares of the error (SSE), sum of absolute error (SAE), average relative error (ARE), average percentage errors (APE), and Marquardt’s percent standard deviation (MPSD) [43].

## 2. Materials and Methods

### 2.1. Formation of Copper Oxide Nanoparticles (CuONPs)

The formation of copper oxide nanoparticles (CuONPs) was performed with the use of precursor copper nitrate hexahydrate (Cu(NO_3_)_2_·6H_2_O), n-propylamine (C_2_H_5_CH_2_NH_2_), surfactant CTAB (cetyltrimethylammonium bromide), and sodium hydroxide (NaOH). All chemicals for the preparation of NPs were purchased from Aldrich Chemical Corporation based in Riyadh, Saudi Arabia and used as received from the company without further purification. In a typical experiment, 15 mM copper nitrate hexahydrate (~0.4425 g) was mixed in 100 mL methanol (MeOH) under constant stirring. The soluble mixture appeared blue in color in a beaker after mixing. To this alcoholic mixture, n-propylamine (20 mL) was added slowly and was dissolved in methanol (MeOH) under continuous stirring at room temperature. A small amount of surfactant CTAB (cetyltrimethylammonium bromide) (5 mg) was added to this solution, which was again mixed. The solution was stirred for 30 min to complete the dissolution. To this solution, alkali sodium hydroxide (NaOH) was added gradually, drop by drop; this mixture was shaken each time for complete mixing. After complete mixing, the solution pH was checked via a pH meter (Cole Parmer, Bunker Court Vernon Hills, IL, USA). Due to the addition of alkali NaOH, the basicity of the solution increased and reached pH 12.06. After complete mixing, the solution was transferred to a two-necked refluxing pot and refluxed at 65 °C for 2 h. As the solution temperature rose, the blue color of the solution changed to a dark brown color and then turned black. After the refluxing was complete, the precipitate of the formed product settled at the bottom of the refluxing pot. The formed precipitate was washed several times with alcohol (methanol (MeOH) and ethanol (EtOH)) and acetone to remove the ionic impurities and then dried at room temperature. The dried powder was examined in terms of its structural and chemical properties.

### 2.2. Characterization of the Prepared CuONPs 

The general morphology of the synthesized CuONPs was examined via field emission scanning electron microscopy (FE-SEM, Hitachi S-4700, Tokyo, Japan) at room temperature. For the detailed observation by FE-SEM, the dried powder was uniformly scattered on carbon tape and coated/sputtered with a thin conducting layer of platinum (Pt) for 3 s. For clarification related to the morphology of the prepared NPs, the powder was again analyzed with TEM (JEOL JEM JSM 2010 at 200 kV, Tokyo, Japan). For TEM observation, nano-ranged powder was sonicated in ethanol for 10 min and a copper grid (carbon coated 400 mesh, Aldrich Chemical Corporation, Branch based in Riyadh, Saudi Arabia) was dipped into this solution. The copper grid was dried at room temperature and analyzed for further morphological observation. The X-ray diffraction (XRD, Rigaku, Tokyo, Japan pattern was utilized to know the particle size, phase and crystalline property of the precipitated material in the range from 20°–65° in Cu_Kα_ radiation (λ = 1.54178 Å) with 6°/min scanning speed with an accelerating voltage of 40 kV and current of 40 mA. For the formation of NPs, various chemicals were involved for the synthesis of the NPs. 

### 2.3. Photocatalytic Activity of Synthesized CuONPs

The photocatalytic degradation of MB dye in the presence of CuONPs was carried out in a homemade photocatalytic glass reactor as per previously described work [1,5,32,33]. To know the detailed photocatalytic degradation of MB dye, about 5 mg of the prepared CuONPs was added to 1 × 10^−5^ M of MB dye solution in 100 mL distilled water under continuous stirring. The blank or control experiment was also included to show that no other reaction happened in the absence of UV light. In our experiment, the CuONPs and MB dye solution sample (5 mL) were extracted each time to remove the catalyst by ultracentrifugation (3000 rpm/min for 5 min, FLETA 5, Hanil Sciind. Co., Ltd., Gimpo, South Korea) before recording the UV-visible data. The light was exposed on a home made reactor and the UV-visible data was collected at regular intervals (0, 10, 20, 30, 40, 50, and 60 min) [1,5,32,33].

### 2.4. Adsorption Procedure

To perform the adsorption process, a conical flask (100 mL) was used for the MB dye with CuONPs under ambient conditions. Different concentration ranges from 51.17 to 153.52 ppm of MB dye was selected for the experiment. The selected amount of adsorbent (~0.10 g of CuONPs) was added to the MB dye solution concentration under constant stirring at 90 rpm and 303 K at pH 12.0 for 1 h to reach its equilibrium point. The optimized/experimental solution was used at regular time intervals. The sample (dye and adsorbent) was completely mixed, centrifuged (10 min), and the filtrate was collected. The effect of parameters such as pH, time, temperature, adsorbent dose, and dye concentration, were checked in solution phase. The concentrations of MB dye solution were analyzed using a UV-vis spectrophotometer (Shimadzu, Japan, UV-2555) at maximum wavelength (570 nm) and the equilibrium point was calculated by the following expression:

(1) $$q_{t}=\frac{\left(C_{0}-C_{t}\right)\times V}{m}$$

where *q_t_* (mg·g^−1^) is the amount of adsorbed dye per gram of adsorbent, *C*_0_ (ppm) and *C_t_* (ppm) are the concentration in solution at time *t* = 0 and at time *t*, *V* is the volume of the solution (L) and ‘*m*’ is the mass of the adsorbent (g).

#### 2.4.1. Effect of pH on MB Dye Adsorption

The pH range of the buffer solution was optimized and chosen for all the sorption experiments. The different pHs (8, 10, and 12) were applied for the prepared nano-adsorbent in solution form. During the adsorption process, the MB dye uptake by the nano-adsorbent was performed in a conical flask, shaken and rotated at 90 rpm in a water bath with varying temperatures. After centrifugation, the samples were decanted at different time intervals during the equilibrium time period. 

#### 2.4.2. Effect of MB Dye Concentration on Adsorption

Different concentrations (51.17, 102.35, 128.0, and 153.52 ppm) of MB dye were chosen to investigate the effect of concentration of MB dye on the sorption by the nano-adsorbent material. The observed data were defined as the sorption capacity of the adsorbent (*q_t_*) versus time (*t*). The concentration of dye increased in the solution with increased sorption at a constant temperature.

#### 2.4.3. Effect of Adsorbent Dose

The specific amount of nanomaterials used as the adsorbent in the sorption process was selected on quantity levels. Varying the quantity was to designate the skill of the adsorbent dose (0.01 to 0.20 g) to maximally adsorb the MB dye. As well, an increased dose of nano-adsorbent was used to increase the rate of adsorption of MB molecules at a constant temperature. After the optimization process, 0.10 g of adsorbent was fixed for the adsorbate at equilibrium phase.

#### 2.4.4. Effect of Temperature on Adsorption

In the sorption processes, the effect of temperature on the adsorption of MB dye by the nano-adsorbent was evaluated in the equilibrium phase. A range of temperatures (298, 301, and 303 K) was checked with the mixed ingredients of 0.10 g adsorbent and 153.52 ppm adsorbate at 90 rpm and a constant pH of 12 for 80 min. An increased temperature increased the rate of absorption of the absorptive species in a respective manner.

### 2.5. Error Analysis

The analysis of error is to identify an experimental sample solution that reveals adequate results. A minimized value of precision and accuracy provides good experimental error-free data. Hence, appropriate results are monitored from the linearization of isotherm parameters, which observe various regretting error conditions at constant temperature [44,45,46,47,48].

Residual root mean square error (RMSE):

(2) $$\mathrm{RMSE}=\sqrt{\frac{1}{N-2}\overset{N}{\underset{i=1}{\sum }}{\left(qe_{\exp}-qe_{\mathrm{cal}}\right)}^{2}}$$

The Chi-square test:

(3) $$x^{2}=\overset{N}{\underset{i=1}{\sum }}|\frac{{\left(qe_{\exp}-qe_{\mathrm{cal}}\right)}^{2}}{qe_{\mathrm{cal}}}|i$$

The sum of the squares of the errors (SSE):

(4) $$\mathrm{SSE}=\overset{N}{\underset{i=1}{\sum }}{\left(qe_{\mathrm{cal}}-qe_{\exp}\right)}^{2}$$

The average relative error (ARE):

(5) $$\mathrm{ARE}=\;\overset{N}{\underset{i=1}{\sum }}|\frac{\left(qe_{\exp}-qe_{\mathrm{cal}}\right)}{qe_{\exp}}|i$$

The sum of the absolute errors (SAE):

(6) $$\mathrm{SAE}=\overset{N}{\underset{i=1}{\sum }}(qe_{\mathrm{cal}}-qe_{\exp})$$

The average percentage errors (APE):

(7) $$\mathrm{APE}=\overset{N}{\underset{i=1}{\sum }}\;\left|\frac{qe_{\exp}-qe_{\mathrm{cal}}\;/qe_{\exp}}{N}\right|\times 100$$

Marquardt’s percent standard deviation (MPSD):

(8) $$\mathrm{MPSD}=\;100\sqrt{\frac{1}{N-P}\overset{N}{\underset{i=1}{\sum }}\left(\frac{qe_{\exp}-qe_{\mathrm{cal}}}{qe_{\exp}}\right)\begin{array}{c} 2\\ i \end{array}}$$

### 2.6. Equilibrium Isotherm Experiment

In this experiment, 0.10 g nano-adsorbent material and 153.52 ppm MB dye were added to each conical flask and the suspensions were mixed at 90 rpm in a shaker bath at a constant pH of 12 for 80 min. After that, the various concentrations of dye solution that had adsorbed onto the sorbent surface were cautiously determined by a titrimetric technique in solution phase [49,50,51,52,53,54,55].

#### 2.6.1. Kinetic Model

The MB dye was adsorbed onto the surface of the nano-adsorbent, showing maximum affinity toward the ions in solution phase. The kinetic models such as Lagergren’s pseudo-first-order kinetic model, Ho and McKay pseudo-second-order kinetic model, Elovich model, and intraparticle diffusion were supported and the control nano-adsorbent-dye reaction mechanism was performed at varying concentrations of MB dye solution (ppm) at different temperatures (K). The equations of the kinetic models are set in experimental data and obtained best-fit results with a good agreement [56,57,58].

#### 2.6.2. Thermodynamic Study

To investigate the optimized thermodynamic parameters of the chemically synthesized nano-adsorbent used as the adsorbent for the sorption of MB dye, the CuONPs were employed with the dye (MB, 153.52 ppm) in a specialized conical flask and the solution was mixed under stirring. Thereafter, it was separated via centrifugation. After the parameters were optimized, the uptake amount of MB dye at equilibrium 𝑞_𝑒_ (mg·g^−1^) was evaluated and calculated [57].

#### 2.6.3. *Lagergren’s Pseudo-First-Order Kinetics*

The pseudo-first-order equation is generally expressed as follows:

(9) $$d_{\frac{q_{t}}{dt}}=K_{F}\left(q_{e}-q_{t}\right)$$

where *q_t_* is the amount of adsorbate adsorbed (mg·g^−1^) at time *t*, *q*_e_ is the adsorption capacity at equilibrium (mg·g^−1^), *K*_F_ is the pseudo-first-order rate constant (min^−1^), and “*t*” is the contact time (min). Integrating Equation (1) with the initial condition *q_t_* = 0 at *t* = 0 and *q_t_* = *q_t_* at *t* = *t*, the equation becomes:

(10) $$\log\left(q_{e}-q_{t}\right)=\log q_{e}-\frac{K_{F}\;t}{2.303}$$

A plot of log (*q*_e_ − *q_t_*) versus time *t* gives the values of *K*_F_ and *q*_e_ from the slope and intercept, respectively.

#### 2.6.4. Ho and McKay Pseudo-Second-Order Kinetic Model

The pseudo-second-order model is represented as:

(11) $$d_{\frac{q_{t}}{dt}}=K_{s}\;{\left(q_{e}-q_{t}\right)}^{2}$$

where *K*_s_ is the pseudo-second-order rate constant (g·mg^−1^·min^−1^). Integrating Equation (4) and applying the boundary conditions *q_t_* = 0 at *t* = 0 and *q_t_* = *q_t_* at *t* = *t*, the equation becomes:

(12) $$\frac{t}{q_{t}}\;=\;\frac{1}{K_{s}q_{e}{}^{2}}+\;\left(\frac{1}{q_{e}}\right)t$$

A plot of *t/q_t_* versus *t* gives the values of the pseudo-second-order constant *K*_s_ (g·mg^−1^·min^−1^) and *q*_e_ (mg·g^−1^). The initial sorption rate *h* (mg·g^−1^·min^−1^) at *t*→0 is defined as:

(13) $$h=K_{s}q_{e}{}^{2}$$

From Equations (4) and (5), we get the following new equation:

(14) $$\frac{t}{q_{t}}\;=\;\frac{1}{h}+\;\left(\frac{1}{q_{e}}\right)t$$

The initial sorption rate *h* is obtained from the intercept since *q*_e_ is known from the slope; the second-order rate constant *K*_s_ can be determined from the value of *h*.

#### 2.6.5. The Elovich Equation

This equation was validated via chemisorption and it covers the extensive absorptions sites. The equation is as follows:

(15) $$\frac{dq_{t}}{d_{t}}=\;\alpha e^{-\beta q_{t}}$$

Integrating this equation for the boundary conditions gives:

(16) $$q_{t}=\;\frac{1}{\beta }\ln\left(\alpha \beta \right)+\frac{1}{\beta }\ln t$$

where *α* (mg·g^−1^·min^−1^) is the initial sorption rate and *β* (g·mg^−1^) is related to the extent of surface coverage and the activation energy involved in the chemisorption.

#### 2.6.6. Intraparticle Diffusion Equation (IDE)

The IDE is as follows:

(17) $$q_{t}=\;K_{\mathrm{id}}.t^{1/2}+\;I$$

where *K*_id_ (mg·g^−1^·min^−1/2^) is the intraparticle diffusion rate constant. A plot of *q_t_* versus *t*^1/2^ presents the adsorption of dye onto the adsorbent. Values of *I* give an idea of the thickness of the boundary layer; that is, the larger the intercept, the greater is the boundary layer effect. 

### 2.7. Equilibrium Isotherm Models

Isotherm models represent the relationship between the concentration of the adsorbate (MB dye) solution in parts per million (ppm) and the amount of dye adsorbed. To evaluate this, various chemical equations were applied, such as Freundlich, Langmuir, Dubinin–Radushkevich, and the Temkin isotherm, to define the equilibrium features of adsorption.

#### 2.7.1. The Langmuir Adsorption Isotherm

The Langmuir adsorption isotherm is represented by the following equation:

(18) $$\frac{1}{q_{e}}=\left(\frac{1}{K_{L}q_{m}}\right)\frac{1}{C_{e}}+\;\frac{1}{q_{m}}$$

where *C*_e_ (ppm) and *q*_e_ (mg·g^−1^) are the equilibrium concentrations in the liquid and solid phases, respectively; *K*_L_ and *q_m_* are the Langmuir constants. *q_m_* expresses the maximum adsorbent uptake (mg·g^−1^) and *K*_L_ is related to the energy of adsorption and affinity of the sorbent. The value of *q_m_* and *K*_L_ can be calculated from the plot of 1/*q*_e_ versus 1/*C*_e_. A further analysis of the Langmuir equation can be made on the basis of a dimensionless equilibrium parameter *R*_L_, also known as the separation factor:

(19) $$R_{L}=\frac{1}{1+K_{L}C_{0}}$$

where *K*_L_ is the Langmuir constant and *C*_0_ is the initial metal ion concentration (ppm). It gives favorable adsorption when 0 < *R*_L_ < 1.

#### 2.7.2. Freundlich Adsorption Isotherm

The Freundlich adsorption isotherm can be represented by the following equation:

(20) $$\ln q_{e}=\ln K_{F}+\frac{1}{n}\left(\ln C_{e}\right)$$

where *q*_e_ is the metal ion adsorbed at equilibrium time, *C*_e_ is the equilibrium concentration of CuONPs in solution, *K*_F_ (L·g^−1^) and *n* are Freundlich constants characteristics of the system and indicating the adsorption capacity and adsorption intensity, respectively. The isotherm constant can be calculated from the intercept and slope of a plot of ln*q*_e_ versus ln*C*_e_.

#### 2.7.3. Dubinin–Radushkevich Adsorption Isotherm

The Dubinin and Radushkevich or D–R equation is written as follows:

(21) $$\ln q_{e}=\ln q_{m\;}-\beta e^{2}$$

where *q_m_* is the D–R constant and *ε* is correlated to temperature as follows:

(22) $$e=RT\ln\;\left(1+\frac{1}{C_{e}}\right)$$

The constant *β* gives the mean free energy *E* of sorption per molecule of sorbate when it is transferred to the surface of a solid from the bulk solution and can be computed using the following relationship:

(23) $$E=1/\sqrt{2\beta }$$

The graph is plotted as ln*q*_e_ versus e^2^. The value of *β* and *q_m_* are calculated from the slope and intercept, respectively.

#### 2.7.4. Temkin Isotherm

This isotherm has influences and it clearly takes into consideration the adsorbing species–adsorbent interactions.

The Temkin isotherm is given as follows [43]:

(24) $$q_{e}=\frac{RT}{b}\ln\left(K_{T}C_{e}\right)or\;B_{1}\ln K_{T}+\;B_{1}\ln C_{e}$$

where

(25) $$B_{1}=\frac{RT}{b}$$

A plot of *q*_e_ versus ln*C*_e_ enables the determination of isotherm constants *B*_1_ and *K_T_* from the slope and intercept, respectively. ‘*R*’ is the gas constant, ‘*b*’ is Temkin constant, *K_T_* is the equilibrium binding constant (L·g^−1^) corresponding to the maximum binding energy. The constant *B*_1_ is related to the heat of adsorption.

#### 2.7.5. Harkins–Jura Isotherm

The multilayer adsorption and the existence of heterogeneous pore distribution in the surface of the adsorbent are mainly described by the Harkins–Jura isotherm model, which is expressed as

(26) $$\frac{1}{q_{e}{}^{2\;}}=\;\frac{B_{\mathrm{HJ}}}{A_{\mathrm{HJ}\;}}-\frac{1}{A_{\mathrm{HJ}}}\;\log\;C$$

where *A*_HJ_ and *B*_HJ_ are Harkins–Jura constants. The value of *A*_HJ_ and *B*_HJ_ are determined from the slope and intercept, respectively, of the plot of 1/*q*_e_^2^ versus log*C*_e_.

#### 2.7.6. Hasley Isotherm Model

Hasley isotherm can be used to evaluate the multilayer adsorption process at a relatively large distance from the surface. The model can be expressed as:

(27) lnqe=1nHlnKH−1nHln(1 Ce)

where $n_{H}$ and *K*_H_ are the Hasley constants, which can be determined from the slope and intercept of the linear plot based on ln*q*_e_ versus ln(1/*C*_e_).

### 2.8. Thermodynamic Study

The change in Gibbs free energy for the adsorption is related to the equilibrium constant by the classic van’t Hoff equation:

(28) ΔG=−RTlnK

This Gibbs free energy change is directly related to the entropy change adsorption heat at constant temperature and can be presented as follows:

(29) ΔG=ΔH−TΔS

(30) $$log\frac{q_{e}}{C_{e}}=\frac{\Delta S}{2.303R}+\left(\frac{-\Delta H}{2.303RT}\right)$$

where *q*_e_ is the dye adsorbed (mg·g^−1^), *C*e is the equilibrium concentration (ppm), *T* is the temperature (Kelvin), and *q*_e_/*C*_e_ is called the adsorption affinity. The above equation is for unit mass of adsorbent dose.

## 3. Results and Discussion

### 3.1. Structural Morphological Description of Prepared CuONPs (FE-SEM and TEM Results)

**Figure 1:**
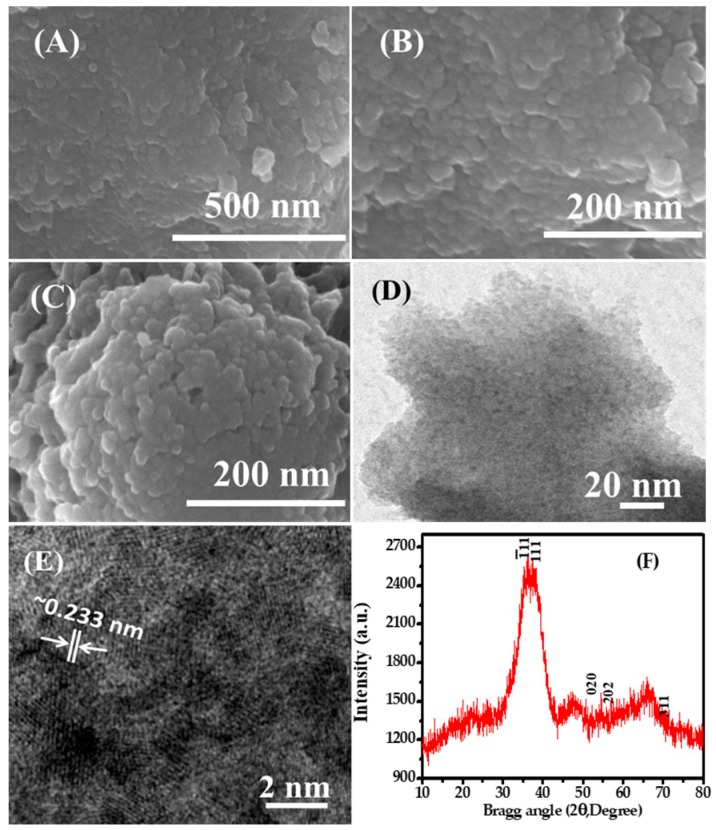
Field emission scanning electron microscopy (FE-SEM) images of copper oxide nanoparticles (CuO NPs): (**A**) NPs at low magnification and (**B**) high magnification; (**C**) After the adsorption of NPs; (**D**) TEM images of CuO NPs at low magnification; (**E**) HR-TEM images of NPs with lattice fringes about ~0.233 nm apart and showing the “d” value of the (111) of CuO NPs; (**F**) X-ray diffraction pattern of grown copper oxide NPs.

The general structural morphology of the chemically prepared NPs was investigated under FE-SEM and the obtained images are presented in Figure 1A,B. From the images, it is visualized that the NPs are in an aggregated Figure 1A form with several fine NPs. When the prepared NPs are viewed at high resolution Figure 1B, it is confirmed that the prepared material is in the nanoscale range and present in a very large quantity. The obtained fine powdery material is spherical in shape and exhibits an average size of ~10–15 nm. Several fine nano-scaled particles are seen in the images; some are spherical and some are in aggregated form. From the image, the surfaces of the NPs are smooth, clean and spherical in structure Figure 1A. Another FE-SEM image Figure 1C shows the adsorption of methylene blue (MB) dye molecules. After the adsorption of MB dye, the image clearly shows that the NPs are joined with each other and form aggregated nanostructures due to the adsorption of organic dye molecules. Further investigation related to structural morphology of the prepared NPs was performed via TEM at room temperature with an accelerating voltage at 200 kV. From the obtained image, it is evident that the small NPs are gathered together and have fine particles in an aggregated form Figure 1D. The diameter of each NP is in the range of ~10–15 nm, is spherical, smooth, and clearly consistent with the observation from FE-SEM Figure 1A,B. The high-resolution TEM image shows that each particle is crystalline in nature, is uniform in size and shape, and has crystalline properties Figure 1E. The HR-TEM image shows the lattice space between two fringes to be ~0.233 nm Figure 1E, which corresponds to the “d” value of the (111) facets of CuO crystals, marked in white lines and arrows in the TEM picture of the CuONPs [59,60,61]. The obtained image is clearly consistent with the FE-SEM observation (Figure 1A,B) [59,60,61].

### 3.2. X-ray Diffraction Spectroscopy (XRD)

The phase, size, and crystalline property of the synthesized (grown) nano-powder were analyzed via XRD at the above parameters. Figure 1F shows a broad peak width, which easily affirms that the size of the grown particles is very small and that the nano-powder has a single crystalline character of CuO without any chemical impurities. It is also well matched with the standard JCPDS data card no. 05-661. The diffraction peaks in the XRD pattern observed at the diffraction reflections appear at 35.55° (Ī11), 38.80° (111), 53.95° (020), 58.40 (202), and 72.40° (311), respectively, which show the XRD spectrum for the formed CuO nanostructures (Figure 1F). The wider width of the peaks clearly shows that the size of the grown nanoparticles is very small. The XRD spectrum shows only peaks related to CuO, without any other impurities or anything other than CuO, which further confirms that the grown powder is pure CuO (Figure 1F).

### 3.3. Catalytic Assessment of CuONPs in the Presence of UV Light 

**Figure 2:**
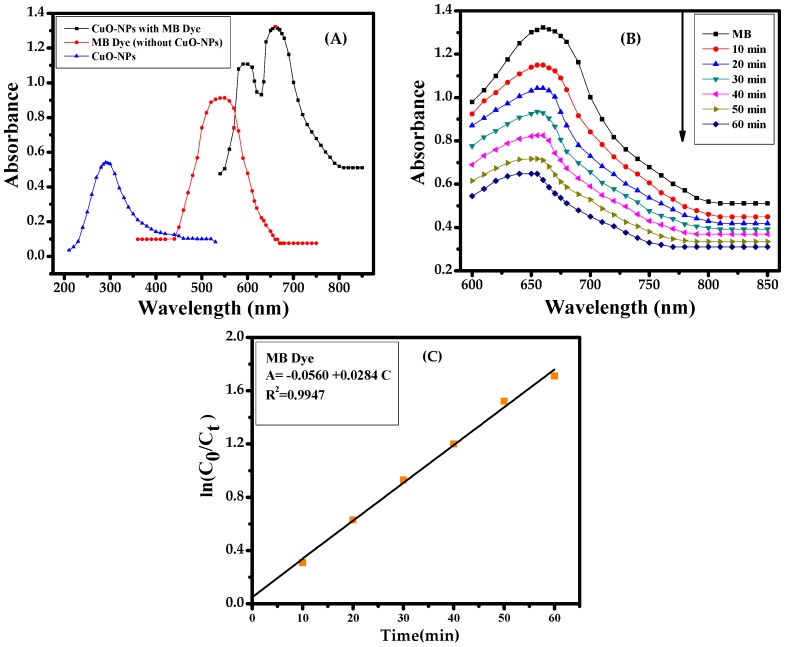
(**A**) UV spectra of methylene blue (MB) dye with CuONPs; (**B**) catalytic reduction of MB dye with CuONPs and (**C**) calibration plot of ln(*C*_0_/*C_t_*) vs. time for the photodegradation of MB dye.

To examine the catalytic sorption/degradation activity in the presence of MB dye under the employed nanomaterial (CuONPs), the initial UV-visible spectroscopy was analyzed with the blank solution of MB dye and thereafter NPs with MB dye Figure 2A. The acquired results show the reduction properties of NPs against MB dye under UV (ultra violet) wave lengths Figure 2B. For further elucidation, kinetic reactions were applied and they follow first-order and reaction rate kinetics as previous Figure 2C [3,4]. The rate constant of CuONPs and MB dye is 2.23 × 10^−2^ min^−1^ and the photocatalytic efficiency is 90.74%.

#### 3.3.1. Effect of pH

**Figure 3:**
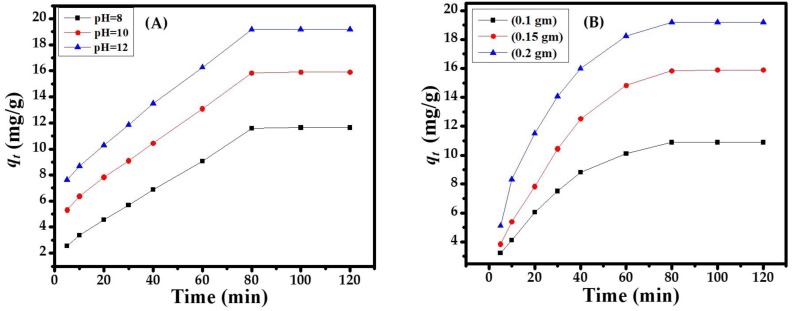
(**A**) Effect of pH on the adsorption of dye; (**B**) Effect of the adsorbent dose on dye adsorption at pH 12.

The great impact of pH on the adsorbent surface encourages the ionization of molecules or the diffusion of ions/functional groups on the active sites of the CuONPs adsorbent, maintaining the surface charge during the adsorption process. The variable pH (8, 10, 12) of the solutions was used in the sorption process and enhanced the sorption activity in a basic medium with pH 12.0. It can be seen in Figure 3A that the maximum and constant adsorption value was found at pH 12.0. An increase in pH increases the sorption capacity that reached a maximum point at 80 min Figure 3A, which is suitable and was selected for the overall experiments. 

#### 3.3.2. Effect of Adsorbent Dose

**Figure 4:**
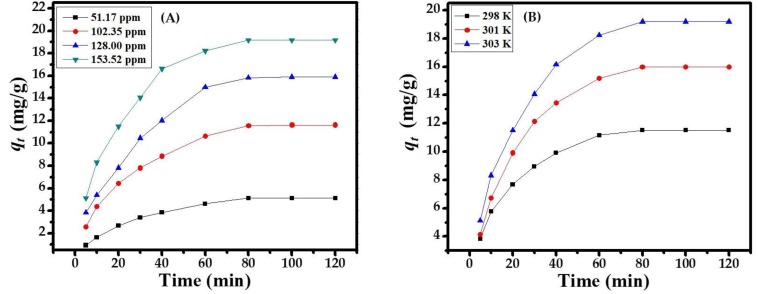
(**A**) Effect of dye concentration at pH 12; (**B**) Effect of dye temperature at 298, 301, and 303 K.

The adsorbent dose was selected by its ability or regulation power to remove/degrade toxic organic compounds (MB dye). Figure 4 shows the variable dose amounts (0.1, 0.15, and 0.2 g). The maximum and constant adsorption capacities were obtained with 0.1 g CuONPs at 303 K in alkaline medium (pH 12.0). The equilibrium concentration in solution phase reached an equilibrium point at 80 min; the amount of the adsorbent dose increases (0.15, 0.2 g) the adsorption capacity, which decreases gradually with a minimum value Figure 3B. Eventually, the adsorbent amount has a large surface area (or active sorbent sites) that takes up the concentration of sorbate molecules. Hence, the per-unit-mass of the sorbent remains constant or increases the sorbent amount, which decreases the amount of sorbate (MB dye). Finally, the potential sites of the adsorbent amount were effective for the intended reduction in the toxic MB dye at the desirable optimization parameters Figure 3B.

#### 3.3.3. Effect of Concentration

The concentration of sorbate (MB dye) ions was impressive on the sorbent surface. The sorbent capacity was determined by the uptake concentration of methyl blue dye (51.17, 102.35, 128, and 153.52 ppm), corresponding to the equilibrium phase. The various concentrations of the MB dye illustrated the highest sorption capacity of CuONPs (153.52 ppm) and the lowest sorption capacity (51.17 ppm) Figure 4A. Hence, increases in the concentration of the MB dye with increases in the sorption capacity at equilibrium point (80 min) gives a maximum and constant sorption capacity at 153.52 ppm, dose at 0.1 g, pH at 12.0, and temperature at 303 K. All the values are optimized and follow photocatalytic and degradation kinetics, isotherm, equilibrium isotherm, and thermo dynamics in an adequate manner.

#### 3.3.4. Effect of Temperature

To study the effect of temperature on the adsorption of MB dye by the adsorbent from a solution of a selected pH, we selected the following temperatures: 298, 301, and 303 K. It is clear from Figure 4B that with an increase in temperature, the adsorption increases, which indicates a chemisorption process. Perhaps this may be due to an endothermic reaction of the dye.

### 3.4. Adsorption Kinetics

The kinetics of the pseudo-first-order equation, pseudo-second-order equation, Elovich equation, and intraparticle diffusion are best fitted with a linear regression equation. The values are summarized in Table S1, which shows that the metal ion adsorption follows the following order:

pseudo-second-order > Elovich model > pseudo-first-order > intraparticle diffusion model

**Figure 5:**
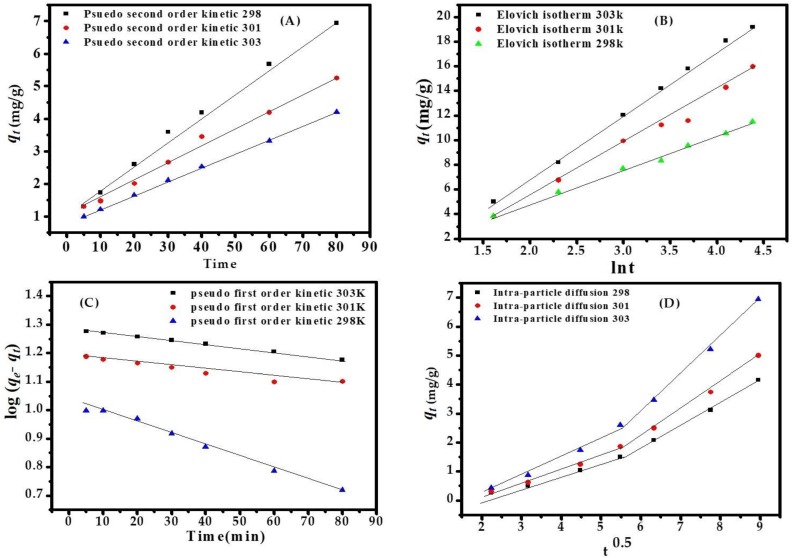
(**A**) Pseudo second order kinetic model (**B**) Elovich kinetic model (**C**) kinetic first order (**D**) Intrapartcle diffusion kinetic model for adsorption of MB dye at various temp eratures.

A plot of *t*/*q_t_* versus *t* gives the value of the constant *K*_s_ (g·mg^−1^·min^−1^); also, *q*_e_ (mg·g^−1^) can be calculated. The descriptive values of the pseudo-second-order kinetics are as follows: *K*_s_ = 0.001454 g·mg^−1^·min^−1^, *q*_e_ = 23.5183 mg·g^−1^, *h* = 1.2431 mg·g^−1^·min^−1^, *R*^2^ = 0.9995. The pseudo-second-order kinetic plot for dye adsorption at a concentration of MB dye of 153.52 ppm, adsorbent dose (CuONPs) of 0.10 g, pH of 12.0, equilibrium at 80 min, and temperature at 303 K, are shown in Figure 5A–D. The value of *K*_f_ = 0.0030 min^−1^ is a pseudo-first-order rate constant and *K*_s_ = 0.0014 min^−1^ is a pseudo-second-order rate constant but the sorption process follows pseudo second order. The Elovich equation—the value of α (2.634 mg·g^−1^·min^−1^) initial sorption rate—covers the extensive absorption sites in Table S2.

### 3.5. Adsorption Isotherm

**Figure 6:**
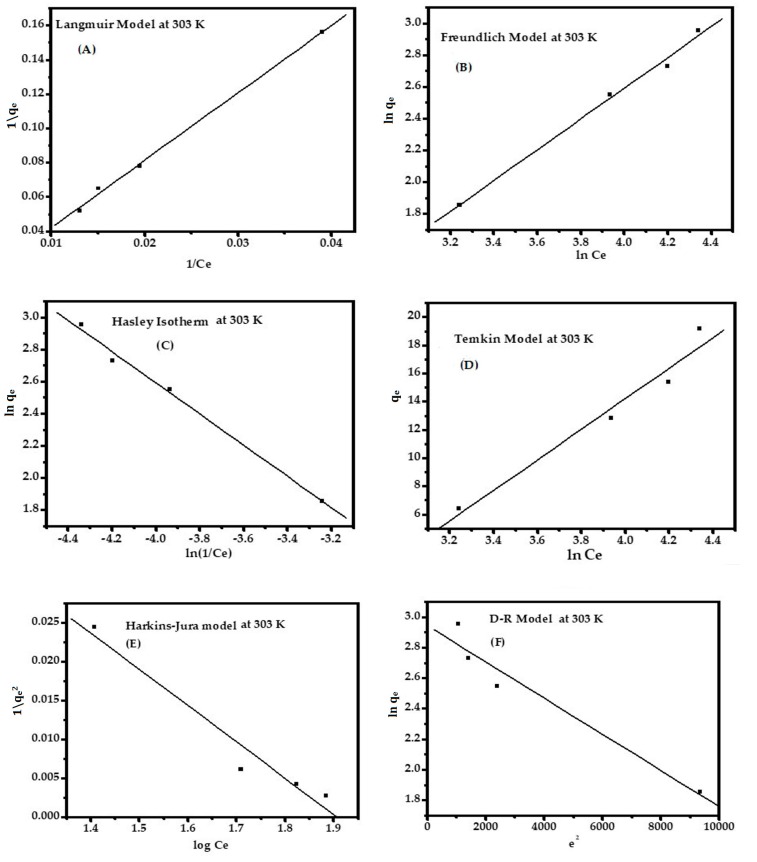
Different types of adsorption isotherm: Langmuir (**A**); Freundlich (**B**); Hasley (**C**); Temkin (**D**); H–J (**E**); and D–R (**F**) for MB dye in pH 12.01 at 303 K.

Adsorption properties and equilibrium parameters, commonly known as the adsorption isotherm, describe how the sorbate interacts with the sorbent surface. The complete consideration of all isotherms help to provide the information about the optimum use of the sorbent. To optimize the design of an adsorption system to organic impurities from dye solutions, it is essential to establish the most appropriate correlation for the equilibrium curve. The sorption can be analyzed via different isotherm equations, especially the Langmuir and Freundlich models, D-R, Temkin, and Hasley. The Harkins–Jura model is infrequently used. All the isotherms described above are shown in Figure 6A–F. The parameters of the isotherms are reported in Table S3 and can be presented with the following order:

Langmuir > Freundlich > Hasley > Temkin > H–J model > D–R model

This can be with the dimensionless separation factor *R*_L_:

(31) $$R_{L}=\frac{1}{1+\;K_{L}C_{0}}$$

where *K*_L_ is the Langmuir constant (L·mg^−1^) and *C*_0_ is the initial concentration (ppm). The value of *R*_L_ determines the isotherm to be unfavorable (*R*_L_ > 1), linear (*R*_L_ = 1), favorable (0 < *R*_L_ < 1), or irreversible (*R*_L_ = 0). It has been found that the range of *R*_L_ is 0.706 to 0.899 for 153.52 ppm at 303 K. The adsorption energy exponentially decreases on the adsorption sites at equilibrium point (*K*_f_ = 0.0.262 mg^1−(1/*n*)^·*L*^1/*n*^·g^−1^) and *n* = 1.030 indicates the strength of the adsorbent for favorable sorption of MB ions. The equilibrium binding constant (*K_T_* = 0.0193), which corresponds to a maximum binding energy and heat of sorption *B*1 = 232.782 J/mol), exhibits a strong interaction between the adsorbate and adsorbent at a constant temperature.

### 3.6. Activation Energy

**Figure 7:**
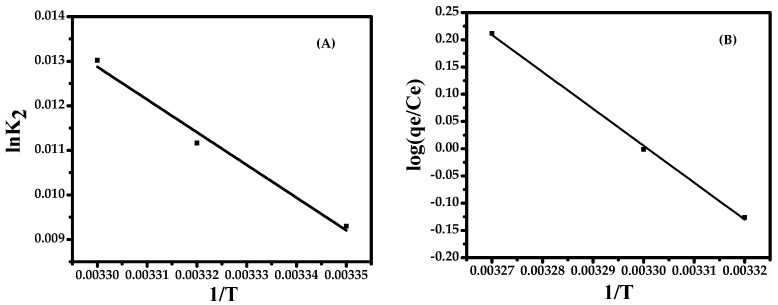
(**A**) The Arrhenius plot and (**B**) the van’t Hoff plot.

The activation energy *E*a is determined according to the Arrhenius equation: lnk_2_ = ln*A*–*E*a/*RT*. A is the Arrhenius constant, the value of *E*a can be determined from the slope of lnk_2_ versus 1/*T* plot (Figure 7A). From the plot, the value of *E*a is 167.87 kJ·mol^−1^. The positive value means sorption process is endothermic in nature and favors sorption with an increase in temperature. 

### 3.7. Thermodynamic Assessment

Temperature dependence of the adsorption process is associated with several thermodynamic parameters. Thermodynamic considerations of the sorption process are necessary to conclude whether the process is spontaneous or not. The values of Gibbs free energy ∆G°, enthalpy change ∆H°, and the entropy of the adsorption ∆S°, which are obtained from the slope and intercept of a plot of log(*q*_e_/*C*_e_) versus 1/*T*, are shown in Figure 7B.

The endothermic nature is also indicated by the increase in the amount of adsorption with an increase in temperature. The positive value of entropy and enthalpy (129,888.183 J/mole and 129,870.009 J/mole, respectively,) indicates that the adsorption process is endothermic in nature and has increased randomness during adsorption. The negative value of ∆G° (−18.1744 J/mol at 303 K) indicates that the reaction is spontaneous.

### 3.8. ErrorAnalysis

The error analysis suggests accurate result values and provides fine values, which differ from the deviation of actual values or true values. In this work, error analysis was performed for the equilibrium isotherm and the types of error details are described along with the isotherms in Table S4. 

## 4. Conclusions

In this work, we synthesized copper oxide nanoparticles via a solution process with the use of copper nitrate hexahydrate (Cu(NO_3_)_2_·6H_2_O), n-propylamine, and sodium hydroxide (NaOH) with surfactant CTAB. The morphology of the NPs was characterized using instruments such as FE-SEM and TEM. The crystalline property of the material was examined with X-ray diffraction pattern (XRD). The obtained nanostructures are spherically shaped with an average diameter of about 10–15 nm, confirmed via TEM images. The photocatalytic performance of the copper oxide nanoparticles was analyzed via UV-visible spectroscopy; their maximum catalytic efficiency (90.74%) for the reduction of methylene blue (MB) dye was achieved in a short time span (80 min). The prepared NPs were employed for the adsorption/degradation of methylene blue (MB) dye in an aqueous medium at pH 12.06. The selected conditions were confirmed via optimization methods for the degradation and adsorption process at maximum and constant adsorbent dose (0.1 g), concentration of MB dye (153.52 ppm), solution pH (12.06), temperature (303 K), and time (80 min) and their equilibrium states. Data analysis of the photocatalytic degradation, kinetic equilibrium, isotherm models, and thermodynamic parameters with error analysis favors the adsorption mechanism. The reliable adsorption data demonstrate that the nanomaterial quantity (0.1 g) allowed the reaction (CuONPs–MB dye) to be possible at the desired optimized reaction parameters. The excellent feasibility is the main selection criteria of the CuONPs with the adsorptive species (conc. 153.52 ppm), activating the adsorption sites for controlled MB dye ion capture due to the maximum adsorption capacity (350.87 mg/g) and the removal efficiency in the equilibrium phase at the highest temperature (303 K). Additionally, the adsorption mechanism is related to behavior properties such as equilibrium adsorption kinetic models, equilibrium isotherm models, and equilibrium thermodynamic parame- ters. The kinetic adsorption provided deeper insight into the structural characteristics and the four models applied, which is the best-fit results decided from the *R*^2^ value (0.9995), describe the adsorption mechanism following pseudo-second-order in the sorption process. The adsorption isotherm models were also selected on the *R*^2^ value. Langmuir models express the best fit straight line graph with an excellent correlation coefficient (*R*^2^ = 0.9988) compared to the other models (D−R = 0.9251). The dimensionless separation factor (*R*_L_) values (0.899) of less than 1 indicate the favorable chemisorption considering the MB ions are uniformly distributed on the adsorbent surface at 303 K temperature. The thermodynamic parameters (∆G°, ∆H°, ∆S°) provide imperative information about the sorption mechanism: the adsorption reaction is spontaneous and endothermic in nature (∆G° = −ve while ∆H°, ∆S° = +ve at the highest temperature 303 K). The collection of all data (∆G° = −18.1744, ∆H° = 129,870.009, ∆S° = 129,888.183 J/mole) from the statistical analyses provide support for the sorption process. The endothermic sorption process expresses an increase in adsorption with an increase in temperature and the value of Gaussian energy (*E*a = 167.87 J/mol) indicates the solute’s activity per molecule of adsorbate on the surface of the adsorbent. The error analysis—such as residual root mean square error (RMSE), Chi-square test, sum of the squares of errors (SSE), average relative error (ARE), and sum of the absolute errors (SAE), average percentage errors(APE), Marquardt’s percent standard deviation (MPSD)—provides various values with respect to the order of significance and addresses the balance issue of the driven sorption mechanism.

## Supplementary Materials

The following are available online at http://www.mdpi.com/2079-4991/8/3/134/s1, Table S1: Adsorption kinetics parameters; Table S2: Linear regression equation fit for adsorption kinetics; Table S3: Adsorption equilibrium isotherm parameters; Table S4: Error analysis for Isotherm models.
