# Supplementary material for: Nanotransition Materials (NTMs): Photocatalysis, Validated High Effective Sorbent Models Study for Organic Dye Degradation and Precise Mathematical Data’s at Standardized Level
Source: Nanomaterials (Basel). 2018 Feb 27;8(3):134. doi: 10.3390/nano8030134 (PMC5869625; doi:10.3390/nano8030134)
Supplement: Supplementary file 1 [file nanomaterials-08-00134-s001.pdf]

Supplementary informations

Table S1: Adsorption Kinetics parameters

Temperature	qe_{exp} (mg g^{-1})	Pseudo first order			Pseudo second order			
		k_1 (min^{-1})	qe_{cal} (mg g^{-1})	R^2	k_2 (min^{-1})	qe_{cal} (mg g^{-1})	h (mg g^{-1} min^{-1})	R^2
298K	11.5146	0.0091	10.7894	0.9856	0.006133	13.2415	0.9298	0.9933
301K	15.9925	0.0020	14.7231	0.9376	0.002970	18.6428	0.9685	0.9907
303K	19.1910	0.0030	19.0107	0.9936	0.001454	23.5183	1.2431	0.9995

Elovich	α (mg/g min)	β (g/mg)	R^2	Intraparticle Diffusion	k (mg g^{-1} $min^{-1/2}$)	I (mg/g)	R^2
298K	2.2234	0.3675	0.9956	298K	2.2593	0.9664	0.9628
301K	2.0626	0.2373	0.9851	301K	1.6295	0.6964	0.9626
303K	2.6344	0.1908	0.9986	303K	1.3677	0.5798	0.9597

Table.S2: Linear regression equation fit for adsorption kinetic

System parameter	k_2(g/mg min)	q_e (mg/g)	h (mg/g min)	R^2
Effect of **pH** for 153.52 ppm at 303 K.				
8	0.0111	11.9246	0.6287	0.9868
10	0.0056	13.2205	0.9819	0.9916
12	0.0014	23.5183	1.2431	0.9995
Effect of adsorbent **dose** for 153.52 ppm at 303 K.				
0.1g	0.0014	23.5183	1.2431	0.9995
0.15g	0.0053	14.4237	0.9024	0.9953
0.2g	0.0168	11.8343	0.4236	0.9827
Effect of Temperature for 153.52 ppm.				
298 K	0.0056	13.2205	0.9816	0.9961
301 K	0.0026	19.6889	1.0311	0.9922
303 K	0.0014	23.5183	1.2431	0.9995

Initial Dye concentration at 303K

51.17 ppm	0.0891	7.3051	0.2101	0.9994
102.35 ppm	0.0135	10.6997	0.6467	0.9991
128.0 ppm	0.0075	12.5015	0.8525	0.9923
153.52 ppm	0.0014	23.5183	1.2431	0.9995

Table S3: Adsorption equilibrium isotherm parameters

Isotherm	**Temperature**	**Parameters**		
		**K_F**	**n**	**R^2**
	298	0.5127	1.47732	0.98436
**Freundlich**	301	0.28904	1.1070	0.9909
	303	0.26242	1.03032	0.9969

Isotherm	**Temperature**	**Parameters**		
		**K_L (L mg^{-1})**	**q_m (mg g^{-1})**	**R^2**
	298	0.008109	25.6673	0.9965
**Langmuir**	301	0.001571	136.61	0.9972
	303	0.007248	350.877	0.9988

Isotherm	**Temperature**	**Parameters**		
		**K_T**	**b**	**R^2**
	298	0.0857	469.45	0.9882
**Temkin**	301	0.0673	282.242	0.9979
	303	0.0193	232.782	0.9426

Isotherm	Temperature	Parameters			
		**q_m**	**β**	**E (J/mol)**	**R^2**
	298	11.7970	1.34301×10^{-4}	61.0162	0.9709
**D-R**	301	16.5808	1.4059×10^{-4}	59.635	0.9780
	303	18.9891	1.18781×10^{-4}	64.880	0.9251

**Isotherm**	**Temperature**	**Parameters**		
		**A_{HJ}**	**B_{HJ}**	**R^2**
	298	16.7336	2.0948	0.9453
**H-J**	301	18.2049	1.9632	0.9290
	303	21.3857	1.9065	0.9314
**Isotherm**	**Temperature**	Parameters		
		**K_H**	**n_H**	**R^2**
	298	0.3728	1.4775	0.9843
**Hasley**	301	0.2522	1.1054	0.9912
	303	0.2616	1.0307	0.9909

Table S4 Error analysis for Isotherm models.

S. No	Error analysis for 153.52 ppm at 303 K

1. Langmuir Isotherm

RMSE	X^2	SSE	ARE	SAE	APE	MPSD
0.492	0.0261	0.484	0.0362	0.695	0.906	13.462

2. Freundlich Isotherm

RMSE	X^2	SSE	ARE	SAE	APE	MPSD
0.424	0.0193	0.359	0.0312	0.599	0.781	12.498

3. Temkin Isotherm

RMSE	X^2	SSE	ARE	SAE	APE	MPSD
0.915	0.0930	1.677	0.067	1.295	1.687	18.368

4. D-R Isotherm

RMSE	X^2	SSE	ARE	SAE	APE	MPSD
1.664	0.257	5.540	-0.122	-0.253	-3.066	24.763

5. Halsey Isotherm

RMSE	X^2	SSE	ARE	SAE	APE	MPSD
0.399	0.030	0.319	0.029	0.565	0.736	12.139

6. <u>H-J Isotherm</u>

RMSE	X^2	SSE	ARE	SAE	APE	MPSD
13.273	836.702	352.310	0.978	18.769	24.451	69.930
